# Concept analysis of community health outreach

**DOI:** 10.1186/s12913-020-05266-7

**Published:** 2020-05-13

**Authors:** Hye Young Shin, Ka Young Kim, Purum Kang

**Affiliations:** 1 National Cancer Control Institute, National Cancer Center, 323 Ilsan-ro, Ilsandong-gu, Goyang-si, 10408 Gyeonggi-do Republic of Korea; 2 College of Nursing, Baekseok Culture University, 1 Baekseokdaehak-ro, Dongnam-gu, Cheonan-si, 31065 Chungcheongnam-do Republic of Korea; 3 Department of Nursing, College of Nursing, Gachon University, 191 Hambakmoeiro, Yeonsu-gu, Incheon, 21936 Republic of Korea; 4 College of Nursing, Woosuk University, 443 Samnye-ro, Samnye-eup, Wanju, Jeonbuk 55338 Republic of Korea

**Keywords:** Concept analysis, Outreach, Health promotion

## Abstract

**Background:**

The definition of community health outreach to promote the health of vulnerable populations depends heavily on the particulars of the given health project and community. There is no consistency in the definitions attached to the concept itself. Our study aimed to clarify the general definition of community health outreach to facilitate its understanding and use.

**Methods:**

Walker and Avant’s (2010) method of concept analysis was used to understand community health outreach. A total of 45 articles were included in the analysis after having searched for text on database portals like PubMed, Scopus, CINAHL complete and EMBASE published between 2010 and 2018.

**Results:**

The defining attributes of the concept of community health outreach were purposive, temporary, mobile and collaboration with community. The antecedents were population facing health risks and awareness of health risks. The consequences were increased accessibility and health promotion.

**Conclusion:**

This study proposed the definition of community health outreach as a temporary, mobile project that involves the collaboration of a community to undertake its purposeful health intervention of reaching a population facing health risks. This definition provides a general understanding of the outreach undertaken by health workers and enables the strong connection between health professionals and community residents.

## Background

Health inequalities receive attention worldwide, but they remain a challenging problem among and within countries [1]. Tailored, multifaceted, community-based strategies are required. Community health outreach provides health-related services to community residents who are at a socioeconomic disadvantage [2]. Typically, this population has been exposed to many health risk factors and has a higher prevalence of cancer and cardiovascular and sexually transmitted diseases compared with the general population [3]. Several studies have reported on the effectiveness of community-based outreach projects in providing customized interventions. Such projects employ community health workers, who are familiar with the community, form multidisciplinary teams to encourage institutional cooperation within the community, or facilitate medical accessibility by approaching directly the individuals at risk [4–6].

Community health outreach generally entails engaging in social work with vulnerable populations to address homelessness, drug abuse, mental disorders, youth problems, and prostitution. However, even basic textbooks on social work rarely mention the definition of outreach, and then, it is not considered on its own terms; it is occasionally presented as “detached,” “street-based,” or “preventive” work [7]. The concept of outreach is seemingly easy to understand, but it is not easy to define. Indeed, the definition of community health outreach depends heavily on a health project’s goals and the community’s context. Researchers tend to focus on specific strategies or activities that provide health services as components of a community project [8]; thus, outreach strategies are strongly tied with the community involved [9, 10]. Several factors influence the process: the outreach staff (“personal factors”), outreach procedures (“process factors”), and the community in which the outreach is taking place (“environment factors”) [11]. Therefore, finding a general definition of outreach has proven challenging.

This study aimed to establish a clear-cut concept of community health outreach by analyzing the activities involved in health-related outreach projects performed in communities.

## Methods

### Concept analysis approach

Concept analysis is a formal, rigorous process by which an abstract concept is explored, clarified, validated, defined, and differentiated from similar concepts to inform theory development and enhance communication [12]. Although there are many approaches for analyzing concepts, Walker and Avant’s (2010) method is often used by novices to conduct concept analyses because it has eight clear steps that derive from the process developed by Wilson [13]. Walker and Avant’s method is the appropriate application for the concept of outreach because this concept is obscure and has not been developed. Although theory can differ from context, the present study was conducted to form a generalized concept of outreach in the health community.

### Literature search

We focused on literature on the promotion of community health. Walker and Avant’s method suggests that an unbiased understanding of a concept should not be limited to nursing or medical literature [12]. Nonetheless, we restricted our search strategy to community health promotion because of the absence of concept analysis of outreach in this particular context. This study was conducted according to the guidelines suggested by the PRISMA group [14]. A review of databases was conducted using database portals like Scopus, CINAHL, PubMed and EMBASE because these were specific to health and health care. Initial search was conducted using the term “community health outreach” and comprised articles from 2010 to 2018 only. Since there was a lack of data related to community outreach activities initially, the broad search term of “community outreach” was then included for the PubMed and EMBASE databases specifically. The reason for limiting the search dates (between 2010 and 2018) was to have gained recognition for community outreach activities in the recent years. Search was limited to viewing full text articles or abstracts in English.

**Fig. 1:**
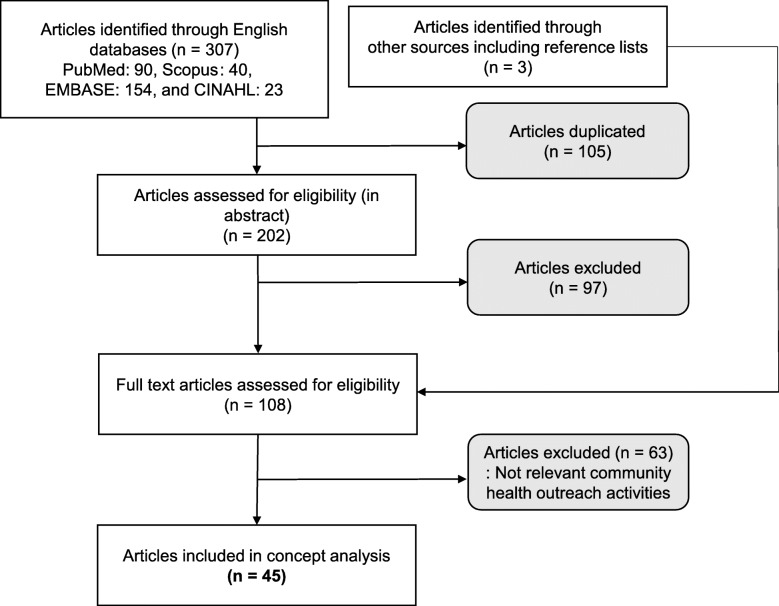
Flow diagram of review process

From the above databases, 307 articles were selected, of which 105 duplicated articles were excluded. Initially, the abstracts were independently reviewed and selected together by two researchers. We discarded at total of 97 articles: protocols, abstracts and those irrelevant to health activities. Selected 105 articles were reviewed in full text and 3 articles were shortlisted from the reference lists of important articles. Then, 63 articles were discarded: focused on need assessments, absence of outreach program description, focused on influencing factor of the outreach program, focused on developing intervention, planning of community outreach. Dictionary definitions were also included. A total of 45 articles were finally included and intensively reviewed in full text. The articles’ retrieval process is displayed in Fig. 1.

## Results

### Identifying use of the concepts from dictionaries

According to *The Oxford English Dictionary* and *Collins English Dictionary*, the word “outreach” has the following definitions:

- An organization’s involvement with or influence in the community, especially in the context of religion or social welfare (*The Oxford English Dictionary*) [15];
- (Social welfare) any systematic effort to provide unsolicited and predefined help to groups or individuals deemed to need it (*Collins English Dictionary*) [16].

There was no definition provided for how outreach relates to health, nursing, or medicine. Therefore, we performed our analysis by identifying outreach programs or projects to arrive at a specific meaning in these fields.

### Identifying uses of the concept based on programs and projects

This study identified the use of the concept of community health outreach using community-based outreach projects or programs as bases.

#### For increasing health screening compliance

The Wisconsin Comprehensive Cancer Control Program strived to increase colorectal cancer screening for undeserved communities. The program developed a partnership with community clinics for engaging the target population by offering screening and follow-ups for community members and hosting outreach events like putting up booths at these clinics, a comprehensive Hispanic health fair, and other special events for invited participants. Approximately 81% attendees received colorectal cancer screenings between 2010 and 2012 [17]. Another outreach program providing a mailing service for fecal occult blood test kits was reported to be an effective strategy for increasing colorectal cancer screening uptakes [18, 19].

The “Helping Her Live” project, which seeks to improve breast screening and follow-ups for abnormal findings, has been implemented among minority women experiencing racial disparities in Chicago since 2008. Community health workers were recruited from target communities to perform outreach interventions and community partnership was built with primary care clinics and mammogram facilities. As a result of outreach activities by community health workers, the rate of mammogram screening completion increased from 35% in 2010 to 72% in 2014 [20].

Brangan, Stone, Chappell, Harrison, and Horwood [21] reported on a telephone outreach intervention conducted to enhance the UK National Health Service (NHS) Health Check program, which provides cardiovascular risk assessment across England every 5 years for individuals who have not been diagnosed with the disease. In this program, those community workers were used who were almost identical to the study participants’ cultural background and language. Seven primary care practices were also engaged to identify eligible patients. These practices successfully engaged a population with higher health needs and reduced health inequalities in the most deprived city of Bristol. Opportunistic outreach services for offering health checks in accessible community venues were conducted in England’s NHS Health Check program. These venues were chosen to collaborate with the community commissioner to reach the target population. These outreach services were more effective in engaging participants in the program than the general physicians [22]. In addition to the above studies, Woringer et al. [23] reported that community health outreach providers offered an effective approach to reach the deprived population for check-ups for cardiovascular disease.

Also, Hoffman, Bryant, Allen, Lee, Aarons, and Kelz [10] reported that a community outreach program involving surgeon engagement was developed, using the Culture, Literacy, Education, Assessment, and Networking (CLEAN) strategy, to address health care disparities in cancer screening and treatment. This program was developed in collaboration with several community-based organizations including Cancer center, Surgical interest group, surgical faculty, and residents within the community. It yielded a unique and synergistic effect for the community’s vulnerable populations.

A Young Parents’ Outreach Center (YPOC) in a large urban Canadian city has provided a full range of health-related services, including pregnancy and nurturing for young pregnant women, fathers and infants; by building community partnerships with family medicine, pediatrics, obstetrics and psychiatry offices. Multidisciplinary outreach clinics have been established in non-clinical settings within the YPOC for the young parent population who require specific medical facilities. Compared with hospital-based mental health clinics, the outreach clinics have reported fewer missed appointments [8]. For the younger population, mental health screening [24] and screening for childhood visual impairment [25] were also conducted.

#### For lifestyle change

The Community Outreach and Cardiovascular Health project was conducted to reduce cardiovascular disease risk factors and change perceptions of chronic illness care in medically underserved areas in [city/state/country]. The project was developed in collaboration with community health centers that were part of Baltimore Medical Systems Incorporated. Nurse practitioners and community health workers’ interventions focused on individualized behavioral services. After 12 months, significant improvements were noted in cardiovascular disease risk factors, including serum cholesterol, blood pressure, and Hemoglobin A1C (HbA1c), compared with the usual care group [26]. The Northern Manhattan Diabetes Community Outreach project was conducted by the community health workers to improve the level of HbA1c, in collaboration with Columbia University’s Medical Center to gather study participants and provide intervention. The intervention yielded modest yet not significant improvements in the HbA1c levels [27]. Also, the Community Outreach Heart Health and Risk Reduction Trial for high-risk subjects with primary or secondary cardiovascular events reported that telehealth lifestyle counseling was more effective at decreasing blood pressure or cholesterol than the recommended guideline for 6 months [28].

The Outreach Pilot Program (OPP) in Puerto Rico was organized to increase smoking cessation. It used community-based participatory research methods in which eight organizations participated within the existing community infrastructures. From 2005 to 2008, the OPP activities included network development, research to build cancer control evidence, training and education for health care professionals, and promotion of community awareness and physician referrals. Physician referrals and the number of annual smokers receiving cessation interventional community services increased significantly [29]. Similarly, the community outreach for smoking cessation in India was conducted [30].

#### For increasing awareness and knowledge

The Healthy Start program, launched by the Community Health Network, developed the “House party model” for outreach and community-based health education to enhance knowledge on maternal and child health in diverse, hard-to-reach populations. The model collaborated with community-based organizations to deliver educational messages on health effectively. It also used community health workers to facilitate and arrange house parties with eight to ten participants. After 23 house parties were held, the participants showed significant improvements in their knowledge base [31].

A community outreach team from the Faculty of Medicine at Ain Shams University conducted hypertension screening among people living in Egypt [32]. This program was facilitated in collaboration with the local health and administrative authorities and data was collected by community workers, a house officer and epidemiologists; from late 2011 to early 2012. The prevalence rates of undiagnosed and uncontrolled hypertension were 11 and 30%, respectively. Finally, they addressed the importance of early detection of hypertension through the community outreach program.

In Ethiopia, a community-based outreach program was conducted for Ethiopian women of reproductive age to improve their knowledge on maternal health and family planning [33]. The project was conducted for 2.5 years in collaboration with the Ethiopian Health Development Army System to recruit study participants. After the project, there was considerable improvement in their knowledge on maternal health and in their behavior. Cofie et al. [9] reported that a community outreach program held in collaboration with a facility-based project called Five Alive, aimed to reduce maternal and child mortality in Ghana. The outreach program built up community collaboration with community leaders to gain access to the communities. They utilized community health workers who performed community-level activities including health education and direct personal-level outreach communication to pregnant women and mothers with children under 5 years of age. In addition to the above, a community outreach program to increase knowledge regarding hepatitis B [34] was conducted.

### Determining the defining attributes

Defining attributes refer to a list of characteristics that “help you and others name the occurrence of a specific phenomenon as differentiated from another similar or related one” (Walker & Avant 2010, p. 162). Reviewing the literature enabled us to identify and generalize the attributes of outreach. The five characteristics that emerged to be most useful in explaining the term “community health outreach” were as follows:

- Purposeful intervention
- Temporary
- Mobile
- Collaboration with community

### Model and additional cases

Examples of model, related, borderline, and contrary cases were used to clarify the concept of community health outreach.

#### Model case

The prevalence of smoking among adolescents in Korea has increased to 30% since 2010. A group of professionals in the field of helping adolescents planned to establish a one-year outreach project to reduce the smoking rate to 20%. Firstly, the project team had collaborated with the middle school authorities and community health centers to facilitate the recruitment of study participants and refer them to health centers in five urban areas. Instructional materials, videos and leaflets were also developed. The project team visited a middle school once a month, to provide smoking cessation education and mailed instructional leaflets regularly. If advanced treatment or counseling were needed, the project team referred individuals to community health centers. Finally, 1 year later, the smoking rate decreased to 10%.

This model case included all the essential attributes and described an ideal community health outreach. The outreach project team provided an intervention for 1 year that included visiting a school to educate the youth and mailed information leaflets to reduce the smoking rate to 20%. It had a purposeful intervention which was temporary and mobile. Collaboration with the community for recruitment and referrals was evident in middle school authorities and community health centers.

#### Related case

The New York City Health and Hospital System is famous for cardiac surgery, and many patients wait a long time for an operation. To reduce the hospital-stay duration of patients, the hospital system launched a home-visit program for patients who were discharged within 3 days of surgery. The program consisted of visits by multidisciplinary teams, including nurses, doctors, nutritionists and physical therapists. They visited patients’ homes to provide services and assess patients’ health conditions. The period and frequency of visiting the homes depended on patients’ conditions and ability to pay. The home visit program resulted in an increase in patient satisfaction and a 75% decrease in waiting time for operation.

This home visit program is similar to community health outreach in a few ways. It had the attributes of purposeful intervention and mobility. The purpose was to provide services and assess patients’ health conditions at home. To reach out to patients, the project team visited patients’ homes, demonstrating movement. However, the home visit program did not specify a project period, and there was no collaboration with the community. Therefore, it was not an example of community health outreach, but it was a hospital-based home visiting care program.

#### Borderline case

The New York City Health and Hospital System is famous for cardiac surgery and many patients wait a long time for an operation. To reduce patient’s stay duration, the hospital system launched a home-visit program for patients who were discharged within 3 days of the surgery. The program consisted of visits by multidisciplinary teams, including nurses, doctors, nutritionists, and physical therapists. They visited patients’ homes to provide services and assess patients’ health conditions. The period and frequency of visiting the homes depended on patients’ conditions and ability to pay. The home visit program resulted in an increase in patient satisfaction and a 75% decrease in waiting time for operation.

This home visit program is similar to community health outreach in a few ways. It had the attributes of purposeful intervention and mobility. The purpose was to provide services and assess patients’ health conditions at home. To reach out to patients, the project team visited patients’ homes, demonstrating movement. However, the home visit program did not specify a project period, and there was no collaboration with the community. Therefore, it was not an example of community health outreach, but it was a hospital-based home visiting care program.

#### Contrary case

A medical appliance manufacturer, SS, developed a new blood glucose test machine. This product was easier to handle at home and was non-invasive compared with older models. After finishing all tests on the accuracy of the machine, SS engaged in promotional activities in a booth setup in front of their office. Whoever wanted to test their blood sugar could use the machine.

This case is contrary to the definition of community health outreach because it had none of the defined attributes. There was no purposeful intervention. Moreover, it did not take a temporary, mobile, or collaborative approach to reach the community. Through promotion activities, SS informed the public of its new product. Therefore, this case was not an example of community health outreach.

### Identifying antecedents and consequences

Walker and Avant explained that antecedents are events or incidents that must occur or be in place prior to the occurrence of the concept, and thus, antecedents cannot also be defining attributes of the same concept [12]. Consequences, meanwhile, are events or incidents that result from the occurrence of the concept; they are the outcomes of the concept (Walker and Avant 2010, p. 167). These antecedents were necessary for the occurrence of community health outreach:

- Populations at health risk
- Awareness of the health risk

A case of community health outreach must have a target population under a health risk. It should outline who needs attention or a special approach. The community health outreach activity is then undertaken based on awareness of a community’s health risks.

Several studies [8–10, 17–22, 35] have shown that community outreach has resulted in increasing levels of public health screenings and accessibility. Other studies [9, 26, 28, 29, 31–33, 36, 37] have reported achieving improved healthy lifestyle changes and increased awareness and knowledge as a result of their provision of services via their community health outreach activities. Thus, the following consequences of community health outreach are supported by our literature review:

Community health outreach results in

- Increased accessibility
- Health promotion (final result)

**Fig. 2:**
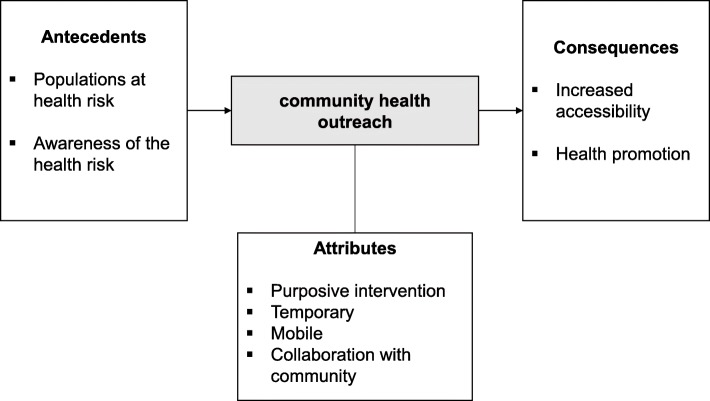
Antecedents, attributes, and consequences of community health outreach

The antecedents, attributes, and consequences of community health outreach are summarized in Fig. 2.

### Empirical referents

Empirical referents are the final step to defining a concept’s attributes. They are classes or categories of actual phenomena that answer the following question: “If we were to measure this concept or determine its existence in the real world, how would we do it?” (Walker and Avant, 2010; p. 168). The present study identified the empirical indicators for each attribute of the community health outreach. First, intervention strategies for achieving the project goals were identified as empirical indicators of purposeful intervention. For instance, the aim of the project Five Alive, was to reduce maternal and child (aged under 5 years) mortality through personal and community-level outreach activities [9]. Various outreach interventions including health education, workshops, employed community health workers etc., aimed to improve compliance with cancer screenings [17–19], lifestyle change [26–30], awareness and knowledge [9, 31–34].

Second, specified operational periods served as empirical indicators to the attribute of temporality. A project for improving maternal health and family planning has been operating for 2.5 years in Ethiopia [33]. The Community Outreach and Cardiovascular Health project, which sought to improve cardiovascular disease risk factors, ran for 12 months [26].

Third, moving towards the target population served as an empirical indicator of mobility. For instance, the outreach staff of a street outreach program went to certain places, such as streets, night clubs and buses, to meet the target audience of young male adults to educate them on sexually transmitted disease prevention methods [35]. Telephonic outreach activities have been shown to enhance health checks [21], and mailing outreach activities to increase colorectal cancer screenings [18, 19]. These methods were used to reach target populations wherever they were.

Fourth, collaboration with a community was identified as community participation, expressing collaboration with experts, systems or leaders in a community. For instance, the project for improving cancer screenings collaborated with a surgical interest group, surgical faculty and surgical residents within a community to recruit study participants and provide the intervention more effectively [10]. The project for imparting knowledge on maternal health and family planning for Ethiopian women was done in collaboration with the Ethiopian Health Development Army System [33].

### Definition

Based on our concept analysis, a community health outreach is defined as follows: A temporary and mobile project that engages the community to collaborate in undertaking its purposeful health intervention to reach the population at health risk.

## Discussion

This study aimed to define the concept of community health outreach by revealing its attributes, antecedents, and consequences using health-related outreach projects performed in communities as bases. The defining attributes of the community health outreach were both essential, including purposive intervention to improve health (increasing health screening compliance, building healthy lifestyle, and increasing health awareness and knowledge), and methodological, including temporariness, mobility, and collaboration of the community. The required antecedents for the occurrence of community health outreach included populations at health risk and awareness of health risks. Increased accessibility and health promotion were among the consequences of the antecedents and project intervention, including the attributes of the community health outreach. Thus, based on our findings, we defined community health outreach as a temporary, mobile project that engages the collaboration of a community to undertake its purposeful health intervention to reach the population at health risk.

Despite continuous efforts at the government level, health inequalities remain, even in developed countries [1]. Community-based heath outreach strategies, which can be applied directly to populations at a socioeconomic disadvantage, have been implemented as among the most effective at resolving health inequalities and building healthy communities [2]. Our findings are expected to contribute precise knowledge on community health outreach to health professionals who play a critical role in establishing community health strategies and providing health-related services. Such knowledge can provide guidance for health professionals in selecting the target community, setting goals, and planning community interventions in detail. In addition, the proposed definition of community health outreach can provide a theoretical basis for health-related disciplines, thereby establishing boundaries with other academic disciplines.

This study had a few limitations. The methods for concept analysis were generally applied to theories, but this study looked at the practice of community health outreach in literature. Second, the articles included in our study were limited to a certain period of time, which may be somewhat limited and may not provide understanding of the community health outreach. However, since the community outreach projects have been actively undertaken since 2010, it will provide in-depth understanding in the context of health. Also, community empowerment is crucial for community health promotion with a final goal of community intervention [38]. However, our study could not identify empowerment as the outcome of the community outreach included in the articles, probably because most outreach projects focused on improvement of urgent health problems or health care to mainly vulnerable populations. Nevertheless, it might provide a basis on concept of community health outreach especially within context of health, and further researches will be continuously conducted to develop and concreate the concept.

## Conclusions

This concept analysis provided a basic definition and identified the antecedents, critical attributes, and consequences of community health outreach from a general perspective. Our proposed definition offers a general understanding of outreach meant to promote community health, and thus, it will enable better communication and facilitate outreach to overcome health inequalities. Future researchers will be able to use this definition to ensure the accurate use of the concept in their studies, whereas health care professionals will be able to identify the antecedents, critical attributes, and consequences of outreach on the ground. As such, our analysis can help guide outreach organizers and health care professionals.

## Supplementary information

**Additional file 1.** The list of articles used for concept analysis on community health outreach.

## Data Availability

The datasets used and/or analyzed during the current study are available from the corresponding author on reasonable request.

## Abbreviations

CINAHL: Cumulative index to Nursing & Allied Health Literature
PRISMA: Preferred Reporting Items for Systematic Reviews and Meta-Analyses
NHS: UK National Health Service
CLEAN: Culture, literacy, education, assessment, and networking
YPOC: Young parents’ outreach center
HbA1c: Hemoglobin A1C
OPP: Outreach Pilot Program
