# Supplementary material for: Concept analysis of community health outreach
Source: BMC Health Serv Res. 2020 May 13;20:417. doi: 10.1186/s12913-020-05266-7 (PMC7222455; doi:10.1186/s12913-020-05266-7)
Supplement: Supplementary file 1 — Additional file 1. The list of articles used for concept analysis on community health outreach. [file 12913_2020_5266_MOESM1_ESM.docx]

**Appendix 1. The list of articles used for concept analysis on community health outreach.**

1. Abd Elaziz KM, Dewedar SA, Sabbour S, El Gafaary MM, Marzouk DM, Aboul Fotouh A, Allam MF. Screening for hypertension among adults: community outreach in Cairo, Egypt. J Public Health (Oxf). 2015;37(4):701-6.

2. Allen JK, Dennison-Himmelfarb CR, Szanton SL, Bone L, Hill MN, Levine DM, West M, Barlow A, Lewis-Boyer L, Donnelly-Strozzo M, et al. Community Outreach and Cardiovascular Health (COACH) Trial: a randomized, controlled trial of nurse practitioner/community health worker cardiovascular disease risk reduction in urban community health centers. Circ Cardiovasc Qual Outcomes. 2011;4(6):595-602.

3. Bang KS, Chae SM, Lee I, Yu J, Kim J. Effects of a Community Outreach Program for Maternal Health and Family Planning in Tigray, Ethiopia. Asian Nurs Res (Korean Soc Nurs Sci). 2018;12(3):223-30.

4. Baur K, Smith T, Wendler MC. What Is It Like to Experience Improved Care Coordination Through a Community Outreach Program? A Qualitative, Descriptive Study. J Ambul Care Manage. 2018;41(3):204-12.

5. Begh RA, Aveyard P, Upton P, Bhopal RS, White M, Amos A, Prescott RJ, Bedi R, Barton P, Fletcher M, et al. Promoting smoking cessation in Pakistani and Bangladeshi men in the UK: pilot cluster randomised controlled trial of trained community outreach workers. Trials. 2011;12:197.

6. Bergman M, Nygren-Brunell O, Vilakati D, Malqvist M. Prolonged Exclusive Breastfeeding Through Peer Support: A Cohort Study From a Community Outreach Project in Swaziland. J Community Health. 2016;41(5):932-8.

7. Castle PE, Rausa A, Walls T, Gravitt PE, Partridge EE, Olivo V, Niwa S, Morrissey KG, Tucker L, Katki H, et al. Comparative community outreach to increase cervical cancer screening in the Mississippi Delta. Prev Med. 2011;52(6):452-5.

8. Chuang E, Wells R, Alexander J, Green S. How outpatient substance abuse treatment unit director activities may affect provision of community outreach services. Drugs (Abingdon Engl). 2013;20(2):149-59.

9. Cofie LE, Barrington C, Akaligaung A, Reid A, Fried B, Singh K, Sodzi-Tettey S, Barker PM. Integrating community outreach into a quality improvement project to promote maternal and child health in Ghana. Glob Public Health. 2014;9(10):1184-97.

10. Diaz-Toro EC, Fernandez ME, Correa-Fernandez V, Calo WA, Ortiz AP, Mejia LM, Mazas CA, Santos-Ortiz Mdel C, Wetter DW. Promoting tobacco cessation and smoke-free workplaces through community outreach partnerships in Puerto Rico. Prog Community Health Partnersh .2014;8(2):157-68.

11. Gueye B, Wesson J, Koumtingue D, Stratton S, Viadro C, Talla H, Dioh E, Cisse C, Sebikali B, Mamadou Daff B. Mentoring, Task Sharing, and Community Outreach Through the TutoratPlus Approach: Increasing Use of Long-Acting Reversible Contraceptives in Senegal. Glob Health Sci Pract. 2016;4 Suppl 2:S33-43.

12. Hilgeman MM, Mahaney-Price AF, Stanton MP, McNeal SF, Pettey KM, Tabb KD, Litaker MS, Parmelee P, Hamner K, Martin MY, et al. Alabama Veterans Rural Health Initiative: a pilot study of enhanced community outreach in rural areas. J Rural Health. 2014;30(2):153-63.

13. Hoffman RL, Bryant B, Allen SR, Lee MK, Aarons CB, Kelz RR. Using community outreach to explore health-related beliefs and improve surgeon-patient engagement. J Surg Res. 2016;206(2):411-7.

14. Johnson D, Harrison P, Sidebottom A. Providing sexually transmitted disease education and risk assessment to disengaged young men through community outreach. Am J Mens Health. 2010;4(4):305-12.

15. Kadaluru UG, Kempraj VM, Muddaiah P. Utilization of oral health care services among adults attending community outreach programs. Indian J Dent Res. 2012;23(6):841-2.

16. Kayama M, Kido Y, Setoya N, Tsunoda A, Matsunaga A, Kikkawa T, Fukuda T, Noguchi M, Mishina K, Nishio M, et al. Community outreach for patients who have difficulties in maintaining contact with mental health services: longitudinal retrospective study of the Japanese outreach model project. BMC Psychiatry. 2014;14:311.

17. LeCheminant JD, Covington NK, Smith J, Lox CL, Kirk EP, Heden TD. Evaluation of a university-based community outreach weight management program. Popul Health Manag. 2011;14(4):167-73.

18. Lynch S, McFarlane WR, Joly B, Adelsheim S, Auther A, Cornblatt BA, Migliorati M, Ragland JD, Sale T, Spring E, et al. Early Detection, Intervention and Prevention of Psychosis Program: Community Outreach and Early Identification at Six U.S. Sites. Psychiatr Serv. 2016;67(5):510-6.

19. Magdalene D, Bhattacharjee H, Choudhury M, Multani PK, Singh A, Deshmukh S, Gupta K. Community outreach: An indicator for assessment of prevalence of amblyopia. Indian J Ophthalmol. 2018;66(7):940-4.

20. Palmas W, Findley SE, Mejia M, Batista M, Teresi J, Kong J, Silver S, Fleck EM, Luchsinger JA, Carrasquillo O. Results of the northern Manhattan diabetes community outreach project: a randomized trial studying a community health worker intervention to improve diabetes care in Hispanic adults. Diabetes Care. 2014;37(4):963-9.

21. Pelton MM. Have fun, be safe: the start of an ED community outreach program. J Emerg Nurs. 2012;38(1):79-80.

22. Riesch SK, Ngui EM, Ehlert C, Miller MK, Cronk CA, Leuthner S, Strehlow M, Hewitt JB, Durkin MS. Community outreach and engagement strategies from the Wisconsin Study Center of the National Children's Study. Public Health Nurs. 2013;30(3):254-65.

23. Salim A, Ley EJ, Berry C, Schulman D, Navarro S, Zheng L, Chan LS. Increasing organ donation in Hispanic Americans: the role of media and other community outreach efforts. JAMA Surg. 2014;149(1):71-6.

24. Venditti EM, Kramer MK. Diabetes Prevention Program community outreach: perspectives on lifestyle training and translation. Am J Prev Med. 2013;44(4 Suppl 4):S339-45.

25. Whitley EM, Main DS, McGloin J, Hanratty R. Reaching individuals at risk for cardiovascular disease through community outreach in Colorado. Prev Med. 2011;52(1):84-6.

26. Salek TP, Katz AR, Lenze SM, Lusk HM, Li D, Des Jarlais DC. Seroprevalence of HCV and HIV infection among clients of the nation’s longest-standing statewide syringe exchange program: A cross-sectional study of Community Health Outreach Work to Prevent AIDS (CHOW). Int J Drug Policy. 2017;48:34-43.

27. Almufleh A, Gabriel T, Tokayer L, Comerford M, Alaqeel A, Kurlansky P. Role of community health outreach program “living for health”® in improving access to federally qualified health centers in Miami-dade county, Florida: a cross-sectional study. BMC Health Serv Res. 2015;15(1):181.

28. Coronado GD, Petrik AF, Vollmer WM, Taplin SH, Keast EM, Fields S, Green BB. Effectiveness of a mailed colorectal cancer screening outreach program in community health clinics: the STOP CRC cluster randomized clinical trial. JAMA Intern Med. 2018;178(9):1174-81.

29. Persell SD, Brown T, Lee JY, Henley E, Long T, Sanchez T, Knight R. Mailed outreach and facilitated test ordering to promote cholesterol screening in community health centers: A randomized trial. J Eval Clin Pract. 2017;23(3):620-4.

30. Liss DT, French DD, Buchanan DR, Brown T, Magner BG, Kollar S, Baker DW. Outreach for annual colorectal cancer screening: a budget impact analysis for community health centers. Am J Prev Med. 2016;50(2):e54-e61.

31. Goldman SN, Liss DT, Brown T, Lee JY, Buchanan DR, Balsley K, Cesan A, Weil J, Garrity BH, Baker DW. Comparative effectiveness of multifaceted outreach to initiate colorectal cancer screening in community health centers: a randomized controlled trial. J Gen Intern Med. 2015;30(8):1178-84.

32. Anderson-Reeves T, Goodman J, Bragg B, Leruth C. House parties: an innovative model for outreach and community-based health education. Matern Child Health J. 2017;21(1):75-80.

33. Brangan E, Stone TJ, Chappell A, Harrison V, Horwood J. Patient experiences of telephone outreach to enhance uptake of NHS Health Checks in more deprived communities and minority ethnic groups: A qualitative interview study. Health Expect. 2019;22(3):364-72.

34. Hunt BR, Allgood KL, Kanoon JM, Benjamins MR. Keys to the successful implementation of community-based outreach and navigation: lessons from a breast health navigation program. J Cancer Educ. 2017;32(1):175-82.

35. Roberts D, De Souza V. A venue-based analysis of the reach of a targeted outreach service to deliver opportunistic community NHS Health Checks to ‘hard-to-reach’groups. Public health. 2016;137:176-81.

36. Norris S, Norris ML, Sibbald E, Aubry T, Harrison ME, Lafontaine G, Gandhi J. Demographic characteristics associated with pregnant and postpartum youth referred for mental health services in a community outreach center. J Can Acad Child Adolesc Psychiatry. 2016;25(3):152.

37. Hamilton J, Sidebottom J. Mountain Pesticide Education and Safety Outreach program: a model for community collaboration to enhance on-farm safety and health. N C Med J. 2011;72(6):471-3.

38. Zimmermann K, Khare MM, Huber R, Moehring PA, Koch A, Geller SE. Southern Seven Women's Initiative for Cardiovascular Health: Lessons Learned in Community Health Outreach with Rural Women. Am J Health Educ. 2012;43(6):349-55.

39. Clifford A, Shakeshaft A, Deans C. Training and tailored outreach support to improve alcohol screening and brief intervention in Aboriginal Community Controlled Health Services. Drug Alcohol Rev. 2013;32(1):72-9.

40. LoConte NK, Weeth-Feinstein L, Conlon A, Scott S. Engaging health systems to increase colorectal cancer screening: community-clinical outreach in underserved areas of Wisconsin. Prev Chronic Dis. 2013;10:E192.

41. Sarkar BK, West R, Arora M, Ahluwalia JS, Reddy KS, Shahab L. Effectiveness of a brief community outreach tobacco cessation intervention in India: a cluster-randomised controlled trial (the BABEX Trial). Thorax. 2017;72(2):167-73.

42. Ruff A, R McFarlane W, Downing D, Cook W, Woodberry K. A community outreach and education model for early identification of mental illness in young people. Adolesc Psychiatry. 2012;2(2):140-5.

43. Dixon S, Fair E, Phillips P, Mansell I, Griffiths C, James M. Promoting annual health checks through community outreach. Learning Disability Practice. 2010;13(6).

44. Zacharias T, Wang W, Dao D, Wojciechowski H, Lee WM, Do S, Singal AG. HBV outreach programs significantly increase knowledge and vaccination rates among Asian pacific islanders. J Community Health. 2015;40(4):619-24.

45. Woringer M, Cecil E, Watt H, Chang K, Hamid F, Khunti K, Dubois E, Evason J, Majeed A, Soljak M. Evaluation of community provision of a preventive cardiovascular programme-the National Health Service Health Check in reaching the under-served groups by primary care in England: cross sectional observational study. BMC Health Serv Res. 2017;17(1):405.
